# Plasmonic Strain Sensors Based on Au-TiO_2_ Thin Films on Flexible Substrates

**DOI:** 10.3390/s22041375

**Published:** 2022-02-11

**Authors:** Marco S. Rodrigues, Joel Borges, Filipe Vaz

**Affiliations:** CF-UM-UP, Centro de Física das Universidades do Minho e do Porto, Universidade do Minho, 4710-057 Braga, Portugal; joelborges@fisica.uminho.pt (J.B.); fvaz@fisica.uminho.pt (F.V.)

**Keywords:** gold nanoparticles, localized surface plasmon resonance, flexible optical sensors, plasmonic thin films

## Abstract

This study aimed at introducing thin films exhibiting the localized surface plasmon resonance (LSPR) phenomenon with a reversible optical response to repeated uniaxial strain. The sensing platform was prepared by growing gold (Au) nanoparticles throughout a titanium dioxide dielectric matrix. The thin films were deposited on transparent polymeric substrates, using reactive magnetron sputtering, followed by a low temperature thermal treatment to grow the nanoparticles. The microstructural characterization of the thin films’ surface revealed Au nanoparticle with an average size of 15.9 nm, an aspect ratio of 1.29 and an average nearest neighbor nanoparticle at 16.3 nm distance. The plasmonic response of the flexible nanoplasmonic transducers was characterized with custom-made mechanical testing equipment using simultaneous optical transmittance measurements. The higher sensitivity that was obtained at a maximum strain of 6.7%, reached the values of 420 nm/*ε* and 110 pp/*ε* when measured at the wavelength or transmittance coordinates of the transmittance-LSPR band minimum, respectively. The higher transmittance gauge factor of 4.5 was obtained for a strain of 10.1%. Optical modelling, using discrete dipole approximation, seems to correlate the optical response of the strained thin film sensor to a reduction in the refractive index of the matrix surrounding the gold nanoparticles when uniaxial strain is applied.

## 1. Introduction

The world market for strain sensors has been thriving in the last decade and is expected to continue growing in the next years, supported by the development of smart cities, buildings automation, and wearable devices [1]. Strain sensors can measure or detect strain, pressure, vibration, impact and deflection on an object after a change in their optical or electrical response. Although most of the commercialized and investigated strain sensors rely on a change in the electrical properties of a material as the transduction mechanism [2,3,4,5], the use of optical phenomena in sensing applications as increased in basic research, health applications and industry, mostly due to its high sensitivity; aging, and high temperature, stability; safety to be used in flammable and explosive atmospheres; and blindness to surrounding electric noise [6,7,8]. Furthermore, the advancements in low-energy optoelectronic components have enabled the construction of miniaturized and portable optical devices [9].

Among the several optical transduction mechanisms, the use of the localized surface plasmon resonance (LSPR) phenomenon, related to the interaction of light with the free electrons of noble metal nanoparticles [10,11,12,13,14,15,16,17,18], has already revealed its capabilities in (bio)chemical sensing [19,20,21,22] and gas sensing [23,24,25,26]. Moreover, regarding the detection of physical and mechanical stimuli, such as temperature and force, some pioneering works have been published, most of them related to theoretical approaches in ultra-thin gold films [27,28,29,30,31,32,33,34,35,36,37,38] and arrays of plasmonic nanoparticles [39,40,41,42,43,44,45,46,47,48]. These and other works led to the development of a new field in plasmonics and nano-optomechanics, also related to mechanochromism [49], named thereafter as plasmomechanics by Maurer et al. [36].

In very simple terms, plasmomechanics studies the reciprocal interactions of light, plasmonic structures, forces applied (from the macro- to the nano-scale) and localized thermal effects [50]. In plasmomechanical systems, the electromagnetic field confinement in metallic nanostructures with subwavelength dimensions, enables to generate large resonance frequency shifts for extremely small displacements. Furthermore, the resonant excitation of these plasmonic nanostructures leads to a strong absorption of the incident light, generating photo-induced thermal effect that can be exploited for actuation of mechanical systems at the nanoscale. Therefore, these systems offer optical and mechanical degrees of freedom for different kinds of applications including active control of nano-mechanical motion transduction and amplification of weak external stimuli [51].

**Figure 1:**
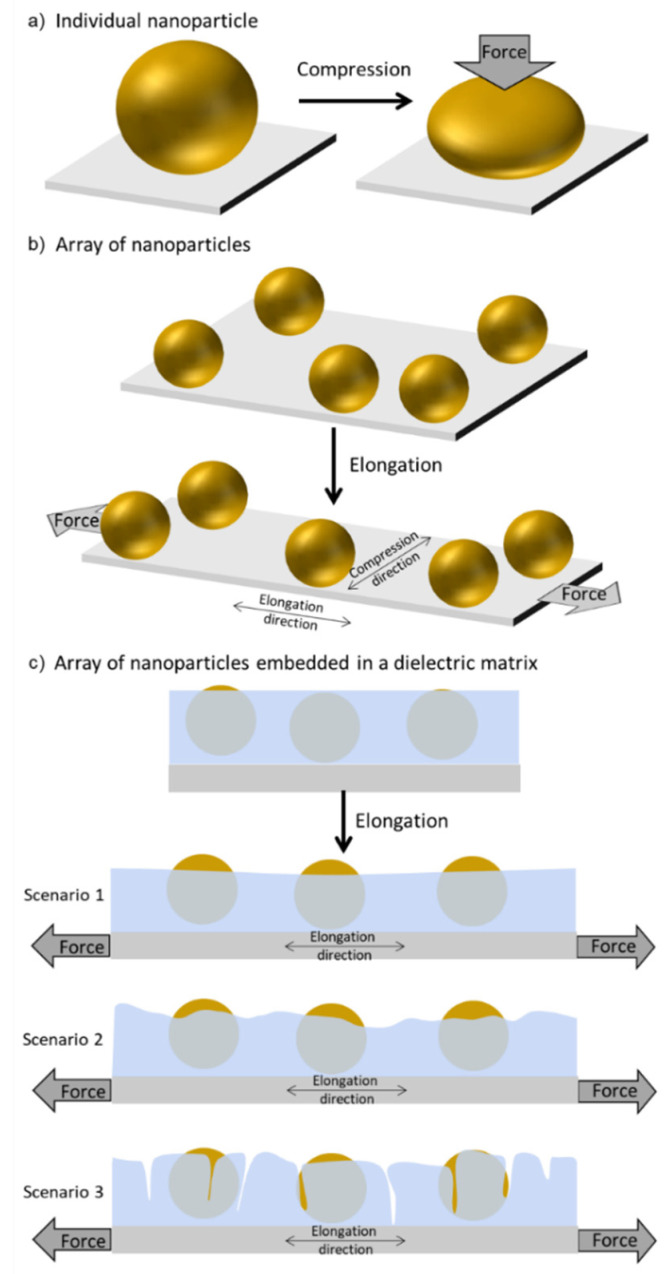
Simplified schematics of deformation mechanisms induced to (**a**) individual nanoparticles being compressed, (**b**) arrays of nanoparticles in a substrate that is being stretched and (**c**) arrays of nanoparticles embedded in a dielectric matrix that is being stretched, where the thickness (scenario 1), the roughness (scenario 2) and the cohesion (scenario 3) of the matrix is changed.

When using plasmomechanics for strain sensing with materials containing noble metal nanoparticles, the transduction mechanism can rely on LSPR band changes induced by external physical stimuli. For example, an applied force may change the nanoparticles’ shape (form) (Figure 1a) and distribution (Figure 1b), or the refractive index (Figure 1c) of the surrounding medium where they might be embedded [36].

In this work, nanoplasmonic thin films with gold (Au) embedded in titanium dioxide (TiO_2_) were deposited on polydimethylsiloxane (PDMS) and characterized. The correlation between the tensile deformation of the flexible nanomaterial and its optical (plasmonic) response was studied. Different mechanisms for strain sensing with nanoparticles exhibiting the LSPR phenomenon were simulated using the discrete dipole approximation (DDA). The obtained results were then used to explain the optical behavior of nanocomposite thin films, when uniaxial strain was applied. The plasmonic sensing platforms’ repeatability was evaluated under several load/unload strain cycles.

## 2. Materials and Methods

**Figure 2:**
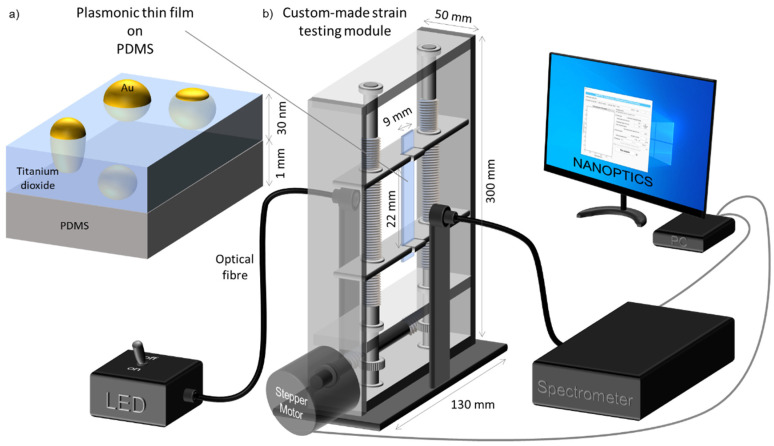
Simplified schematics of (**a**) the sensing platform, a nanoplasmonic thin film (composed by gold nanoparticles dispersed in TiO_2_) deposited on transparent PDMS, and (**b**) the strain testing module coupled to an optical transmittance measurement system.

The sensing platform consists of an optically transparent polydimethylsiloxane (PDMS) substrate that was functionalized with a nanocomposite gold and titanium dioxide (Au-TiO_2_) thin film (Figure 2a). The thin film was deposited by reactive DC magnetron sputtering [52,53]. In order to obtain an optical LSPR response a thermal annealing protocol was applied (150 °C for 10 min) as explained in detail elsewhere [54].

The in-depth chemical composition profile was evaluated by Rutherford Backscattering Spectrometry (RBS). The measurements were conducted in a chamber, where three detectors were installed, a standard at 140° and two pin-diode detectors located symmetrically to each other, both at 165°. Spectra were collected for 2 MeV 4He^+^ beams at normal incidence (0°). The obtained RBS data were analyzed with IBA DataFurnace NDF v10.0a [55,56], and the double scattering and pileup were calculated with the algorithms given in N. P. Barradas et al. [57] and in N. P. Barradas et al. [58], respectively.

To study the microstructure of the thin films, scanning electron microscopy (SEM) was used with a FEI Quanta 650 FEG (Field Emission Gun) environmental SEM microscope operating at 20 kV from INL (International Iberian Nanotechnology Laboratory). Then, a custom MATLAB algorithm was employed to study the features of the Au nanoparticles (Feret diameter, nearest neighbor and aspect ratio) [59].

The plasmonic response of the Au-TiO_2_ thin films was investigated in a custom-made strain testing module coupled with an optical system, as depicted in Figure 2b. 

While the strain module stretches the sensor, its optical transmittance spectrum is measured using a modular spectrometer (SPEC RES + UV/Vis, SARSPEC—with a diffraction grating), and a LED light source (LS-LED, SARSPEC) to excite the localized surface plasmons. Precise strain was controlled by a stepper motor (200 steps per turn) connected to a 20:1 worm reduction gear system, with a total 4000 steps per turn, thus giving the strain movement a resolution of 0.15 µm per step. Two protocols were used to apply stress strains to the sensors, by (i) varying the strain speed keeping the maximum strain constant; and (ii) varying the maximum strain keeping the strain speed constant.

The optical transmittance measurement was synchronized with the applied strain and a full spectrum (from 450 nm to 750 nm) was recorded every 0.5 s with an integration time of 3 ms and an average of 20 spectra. These transmittance spectra data were then analyzed using the software NANOPTICS [60] to follow the LSPR band minimum wavelength and transmittance coordinates. 

Optical modelling and simulation of gold nanoparticles was conducted with the discrete dipole approximation (DDA) method [7,61,62] using the software nanoDDSCAT+ [63] freely available at “nanohub.org” (Figure S1). 

For all the simulations, the extinction spectra were calculated with the following principal parameters: two dipoles per nanometer for each shape; unpolarized incident light; Au dielectric function from Johnson and Christy [64]; surrounding external refractive index set either to 1.5, 2.0, or 2.5, as a “semi-infinite” layer; and 5 nm steps in all spectra windows. For individual nanoparticles with different sizes and aspect ratios, the predefined configurable ellipsoid shapes in DDSCAT tool (Figure S1-III) were used. For the simulations using arrays of nanoparticles, a 16 nm spherical nanoparticle was designed with Blender tool (Figure S1-I), then converted to dipoles with DDA convert tool (Figure S1-II), and exported to DDSCAT tool (Figure S1-III), where, along with the previously described parameters, a 2-D object periodicity was selected, with the *y* and *z* spacings of interest.

## 3. Results and Discussion

### 3.1. Nanoplasmonic Thin Film Characterization

The RBS analysis of the as-deposited thin film revealed a uniform in-depth Au concentration of 22 at.%, across a total estimated thickness of 30 nm.

**Figure 3:**
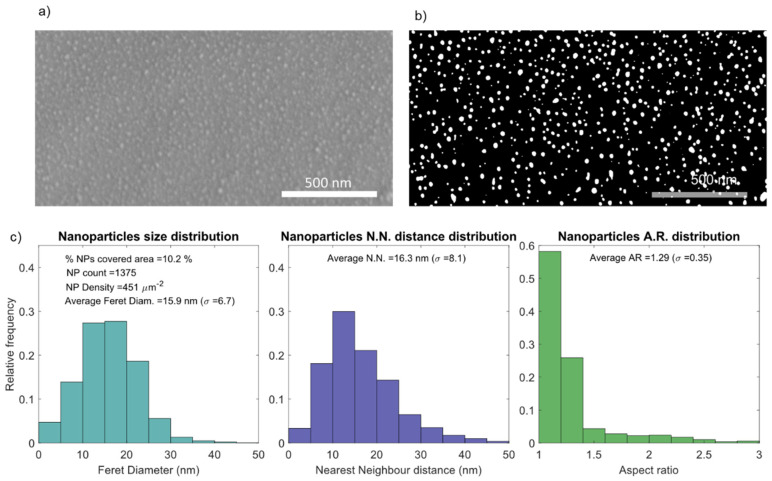
Au nanoparticles analysis in the Au-TiO_2_ thin film deposited on PDMS and annealed at 150 °C, showing the (**a**) SEM top-view micrograph of the Au-TiO_2_ thin film’s surface, (**b**) the thresholded micrograph, revealing the Au nanoparticles in white, as well as (**c**) a statistical analysis of the exposed nanoparticles, with the distribution histograms of nanoparticles diameter (average of 15.9 nm), nearest neighbor distance (average of 16.3 nm) and aspect ratio (average of 1.29).

To study the distribution of Au nanoparticles dispersed in the TiO_2_ matrix, the nanoplasmonic thin film deposited on the flexible substrate and annealed at 150 °C was analyzed using SEM (Figure 3). The micrographs revealed the Au nanoparticles partially embedded in the TiO_2_ matrix, Figure 3a, and equally distributed throughout the observed areas. After image processing in a custom MATLAB algorithm, the SEM micrograph was thresholded to black (TiO_2_ matrix) and white (nanoparticles), as depicted in Figure 3b, thus allowing to analyze the Au nanoparticles distributions, presented in Figure 3c. The nanoparticles’ analysis revealed a 10.2% of area covered by Au, with a nanoparticle density of 451 nanoparticles/µm^2^ and an average size of 15.9 nm. The fact that 55% of the nanoparticles possessed a Feret diameter between 10 and 20 nm is also noteworthy. Regarding the nearest neighbor distance, 51% of the nanoparticles possess a nearest Au nanoparticle neighbor between 10 to 20 nm, giving an average nearest neighbor of 16.3 nm. When analyzing the aspect ratio (AR), an average of 1.29 was obtained, with 58% of the nanoparticles having an AR between 1 and 1.2, and 83% with an AR below 1.4. Following these results, 16 nm Au nanoparticles were used for the starting conditions in the simulations presented in Section 3.2, as well as a starting nearest neighbor distance of 16 nm in Section 3.2.3 and Section 3.2.4. 

### 3.2. Gold Nanoparticles’ Optical Modelling under Different Mechanical Strain Conditions

The LSPR phenomenon arises from the interaction of light with metallic nanoparticles of a size of about one order of magnitude lower than the wavelength of the incident light. This interaction depends on the size, shape and distribution of the nanoparticles [65,66,67] but also on the material surrounding them [59,68], and it can be characterized by studying their extinction spectra, both experimentally and theoretically. In this section, the optical extinction spectra of isolated and arrays of Au nanoparticles was simulated considering the parameters from the previous section, during the application of several mechanical stimulus. The results were then used to be compared to the experimental results in Section 3.3.

Among the numerous methods available for optical modelling, DDA was used because it allows the simulation of nanoparticles with different shapes [7,61] and organized in 1D or 2D networks [62]. 

#### 3.2.1. Compressing Single Nanoparticles

**Figure 4:**
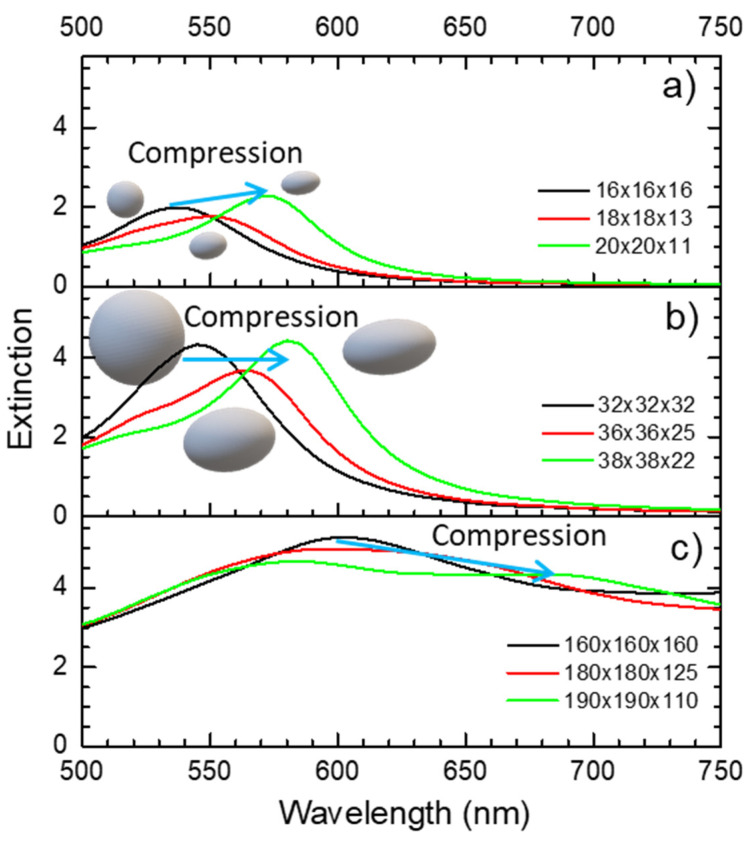
Optical extinction spectra of nanoparticles with different diameters (**a**) 16 nm, (**b**) 32 nm and (**c**) 160 nm, and after being compressed, changing from a sphere (initial state) to an oblate spheroid with an AR of 1.4, and then to an oblate spheroid with an AR of 1.8, calculated with nanoDDSCAT+. The blue arrow shows the shift direction of the extinction spectra (red-shift) when the nanoparticle is compressed.

Nanoparticles with different shapes have distinct optical responses as it can be confirmed in the supplementary material for a Au sphere, an oblate and prolate spheroid, and an ellipsoid with similar effective volumes (Figure S2). While the sphere presents an extinction maximum at a wavelength of 535 nm (when surrounded by a semi-infinite layer with a refractive index of 1.5), for the oblate and prolate spheroid, and for the ellipsoid, the extinction maximum occurs due to the longitudinal vibration modes of the localized surface plasmons [7] that gave rise to a second peak at higher wavelengths, 575 nm, 610 nm and 635 nm, respectively. On the other hand, since a single Au nanoparticle can be deformed elastically [69], if it is compressed and transformed into an oblate spheroid with an AR of 1.4 (as simulated in Figure S3), it gives rise to a second peak in the extinction spectrum at higher wavelengths (Figure 4). As the AR of this oblate spheroid increases to 1.8 (due to the compression of the nanoparticle), the second peak shifts to higher wavelengths. If the maximum compression is limited to the elastic regime of the nanoparticle’s deformation, this process will be reversible; this effect can be used, for e.g., in optical strain sensing.

#### 3.2.2. Biaxial Elongation of Gold Nanoparticles Network

**Figure 5:**
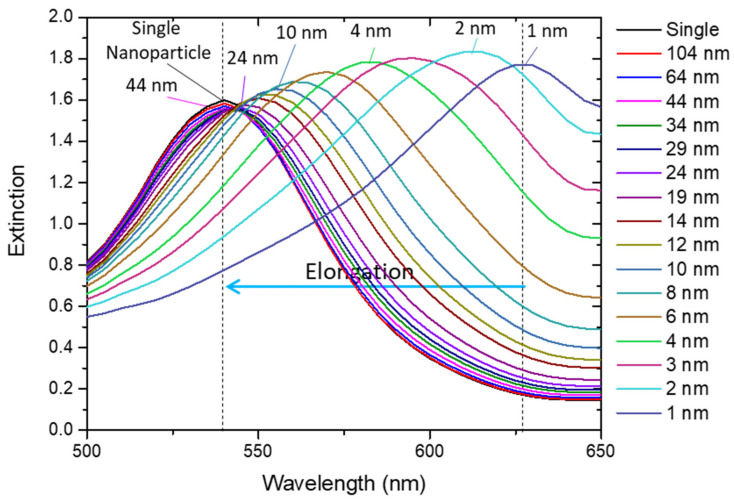
Optical extinction spectra of a network of gold nanoparticles being biaxially elongated, calculated with nanoDDSCAT+. The blue arrow shows the shift direction of the extinction spectra (blue-shift) when the array of nanoparticles is biaxially elongated.

For pairs or arrays of nanoparticles, the LSPR coupling effect between nanoparticles that are close together leads to a strong interaction that can be used for strain and deflection sensing. When considering a biaxial elongation of an array of Au nanoparticles (with 16 nm diameter, for example) where the nearest neighbor starts at a distance of 1 nm, as depicted in Figure S4, the strong coupling effect quickly vanishes, leading to a blue shift of the LSPR band, as can be observed in Figure 5. When the nearest neighbor is at a distance of 24 nm (strain of 135%-*ε* = 1.35), the coupling between the 16 nm nanoparticles is very weak, leading to a total LSPR band shift of 84 nm. Further increasing the nearest neighbor distance from 24 to 44 nm (strain of 253%-*ε* = 2.53) the LSPR band blue-shifts only 3 nm, and the coupling effect practically disappears. In fact, as depicted in Figure 5, the extinction spectrum of the array of nanoparticles for distances higher than 44 nm is similar to the single nanoparticle spectrum. The inverse process (approximation of nanoparticles) would give rise to (an opposite) red-shift.

#### 3.2.3. Uniaxial Elongation of a Gold Nanoparticles Network in Different Surrounding Refractive Indices

For this case, an array of Au nanoparticles with 16 nm diameter, a starting nearest neighbor distance of 16 nm (surface to surface) and surrounded by different refractive indices (1.5, 2.0 and 2.5) was simulated during uniaxial elongation, as depicted in Figure S5. These parameters were chosen considering the nanoparticles’ distribution analyzed in Section 3.1 and previous works from the authors [54,70].

**Figure 6:**
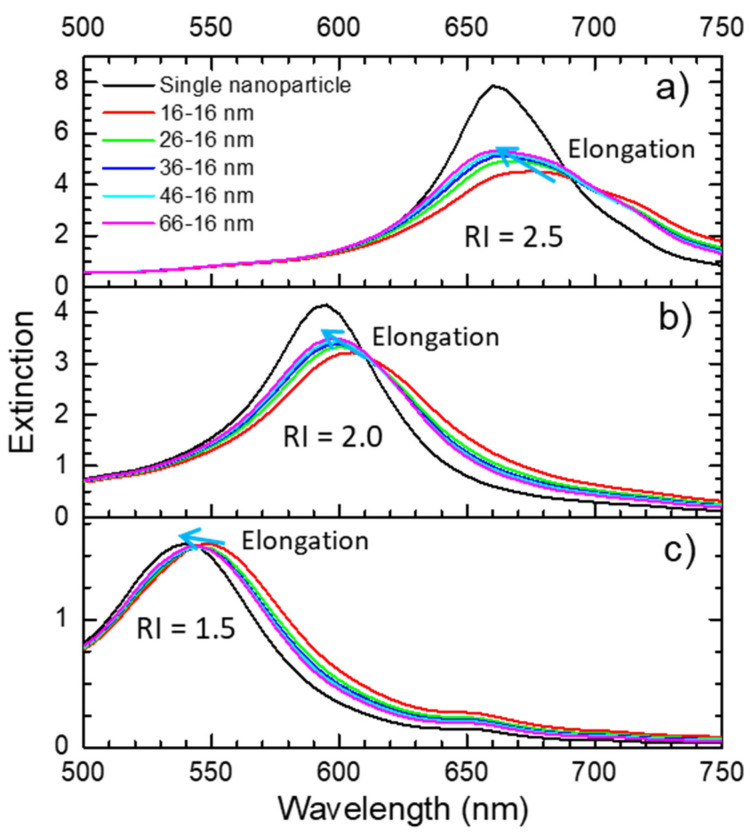
Optical extinction spectra of a network of nanoparticles being uniaxially elongated in a refractive index of (**a**) 2.5, (**b**) 2.0 and (**c**) 1.5, calculated with nanoDDSCAT+. The blue arrow shows the shift direction of the extinction spectra (blue-shift) when the array of nanoparticles is elongated.

From the simulation results, shown in Figure 6, it seems obvious that the refractive index of the material surrounding the nanoparticles holds a strong influence in the position of the LSPR band. In the simple case, i.e., for a single nanoparticle, when the refractive index decreased from 2.5 to 1.5 (Figure 6a,c) it led to a blue-shift of 125 nm. For the arrays of nanoparticles suffering uniaxial elongation (separation in the direction of the elongation), a maximum strain of 156% (*ε* = 1.56) led to blue-shifts of 15 nm, 8 nm and 5 nm for surrounding refractive indices of 2.5, 2.0 and 1.5 (Figure 6a, Figure 6b, Figure 6c), respectively.

Therefore, if one could separate the nanoparticles while lowering the refractive index of the surrounding matrix, a strain sensor with enhanced sensitivity would be obtained.

#### 3.2.4. Elongation of a Gold Nanoparticles Array, Considering Transverse Deformation

Since there is a transverse deformation when a flexible material such as PDMS is elongated in a tensile test, a Poisson’s ratio of 0.499 (from PDMS [71]) was used in the simulations to calculate the elongation and compression of the nanoparticles’ array. Following the previous sections, a similar array of nanoparticles was used (i.e., same initial relative positions) surrounded by a refractive index of 1.5 and a maximum strain of 56% (*ε* = 0.56), as depicted in Figure S6. 

**Figure 7:**
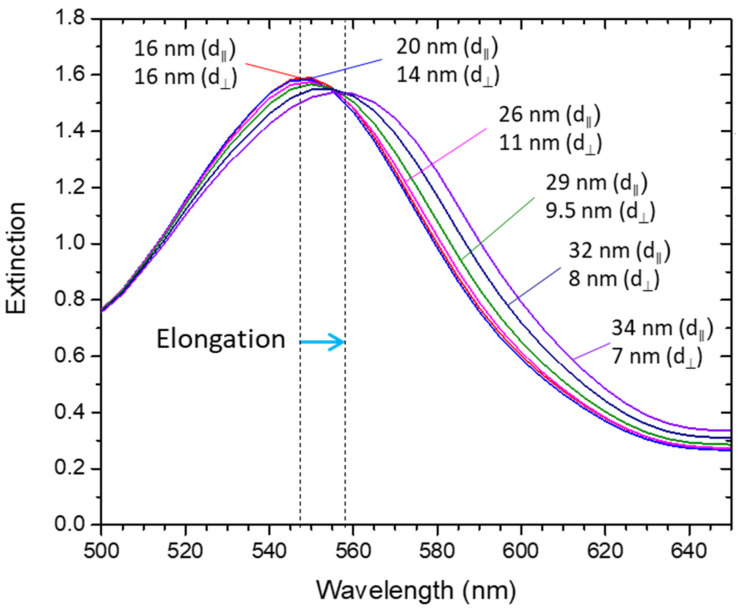
Optical extinction spectra of a network of nanoparticles being uniaxially elongated and transversely compressed, calculated with nanoDDSCAT+. The blue arrow shows the shift direction of the extinction spectra (red-shift) when the array of nanoparticles is elongated while transverse compression occurs.

The extinction spectra presented in Figure 7 indicate that under these conditions, the red-shift of the LSPR band, due to the enhanced coupling between the nanoparticles in the transverse direction to the tensile test direction, overcame the expected blue-shift due to the lower coupling between the nanoparticles in the elongation direction. In this example, applying a maximum strain of 56% (*ε* = 0.56), led to a total LSPR band red-shift of 10 nm.

### 3.3. Experimental Uniaxial Elongation of Nanoplasmonic Au-TiO_2_ Thin Films Deposited on PDMS

**Figure 8:**
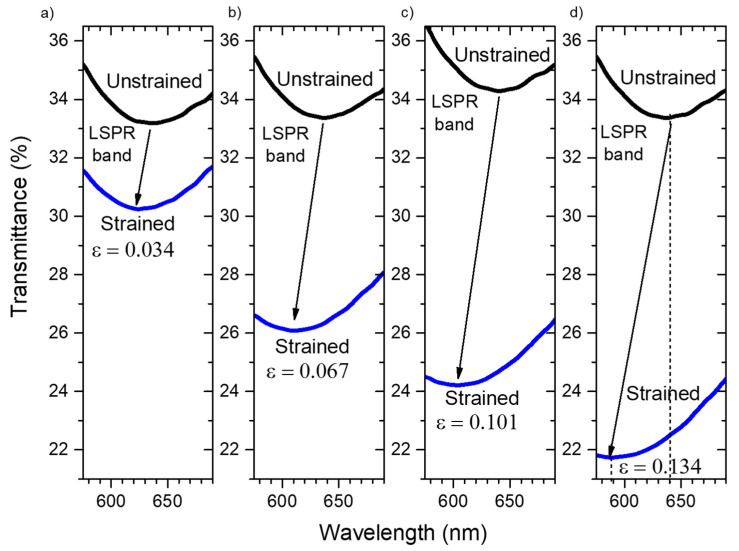
Optical transmittance of the unstrained (black line) and strained (blue line) thin film, with a maximum strain of (**a**) 3.4%, (**b**) 6.7%, (**c**) 10.1% and (**d**) 13.4%. The black arrow shows the shift direction of the extinction spectra (blue-shift and lower transmittance shift) when the nanoplasmonic sensors are strained.

For the experimental tensile tests, a Au–TiO_2_ thin film deposited on flexible and transparent PDMS was used. After a low temperature annealing process at 150 °C for 10 min, the Au nanoparticles were formed (see Section 3.1) and the Au–TiO_2_ thin film revealed its plasmonic nature by exhibiting the LSPR band, as depicted in Figure 8. 

After applying different strains to the flexible sensor, the optical transmittance of the plasmonic sensor suffered an overall reduction, and the LSPR band minimum was blue-shifted, Figure 8. The transmittance coordinate of the LSPR band minimum was reduced by 3 pp, 7 pp, 10 pp and 12 pp, while the wavelength coordinate was blue-shifted by 12 nm, 25 nm, 37 nm and 50 nm, at maximum strains of 3.4% (*ε* = 0.034, Figure 8a), 6.7% (*ε* = 0.067, Figure 8b), 10.1% (*ε* = 0.101, Figure 8c) and 13.4% (*ε* = 0.134, Figure 8d), respectively. 

When comparing the simulation results with this optical LSPR band response to the maximum applied strain of 0.134 (13.4%), it seems that the most important effect is not related to changes in nanoparticle’s coupling. Indeed, and since the results simulated in Section 3.2.4 are the ones with the most comparable conditions, one would expect that the LSPR band would be red-shifting due to a higher coupling effect in the transverse direction [72]. Instead, Figure 8 shows that there was a considerable blue-shift, with the minimum wavelength coordinate varying from 640 nm to 590 nm. 

These results seem to indicate that the refractive index surrounding the nanoparticles might be the main effect, as it can be confirmed by the simulation of the optical behavior evolution in Section 3.2.3.

**Figure 9:**
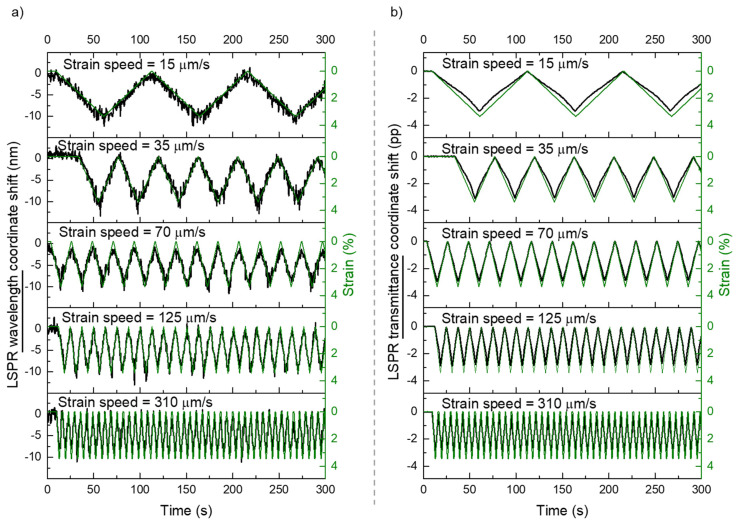
Response of the nanoplasmonic sensor, with the LSPR band minimum (**a**) wavelength and (**b**) transmittance coordinates monitoring during strain cycles at different speeds (from 15 µm/s up to 310 µm/s) and with a maximum strain of 3.4%. In green (right axis) the corresponding strain monitoring for each test.

**Figure 10:**
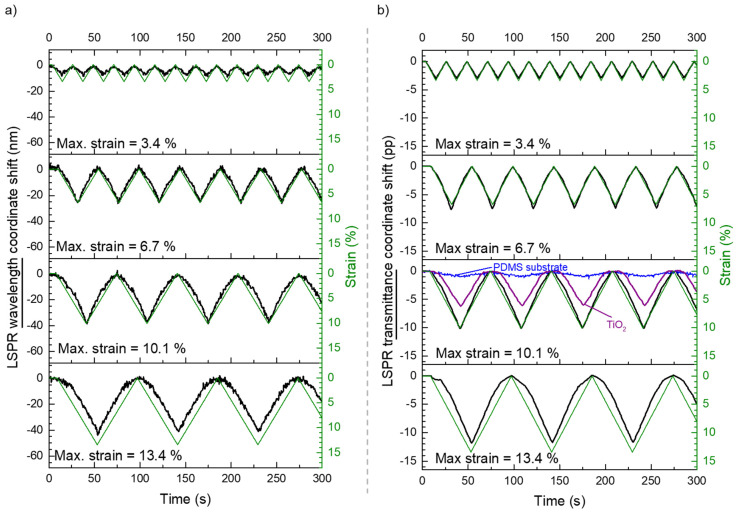
Cyclic response of the nanoplasmonic sensor, with the LSPR band minimum (**a**) wavelength and (**b**) transmittance coordinates monitoring during strain cycles with different maximum strains (from 3.4% to 13.3%) and at a speed of 70 µm/s. In green (right axis) the corresponding strain monitoring for each test. (**b**) also presents in blue and purple, the transmittance at 650 nm of the PDMS substrate and the TiO_2_ thin film deposited on PDMS respectively, for a maximum strain of 10.1% and a strain speed of 70 µm/s.

Following these results, several strain cycles were applied to the same sensor, by changing the strain speed, from 15 µm/s up to 310 µm/s, Figure 9, and the maximum strain, from 3.4% (*ε* = 0.034) up to 13.4% (*ε* = 0.134), Figure 10. The response of the plasmonic thin film sensor was replicated for all the tested conditions with no drifts nor hysteresis in the monitored cycles.

Figure 9 shows the real time response of the plasmonic platform, after consecutive tensile tests with a maximum strain of 3.4% (*ε* = 0.034) by following the variations of the LSPR band wavelength and transmittance coordinates. These results show that the response of the sensor is consistent if the deformation speed is changed, which means that the response time of the sensor is lower, or in the order of the spectra acquisition time (0.5 s). Regarding the quality of the signals, both the wavelength and the transmittance coordinates showed an acceptable signal-to-noise ratio, although the transmittance signal is fairly better.

To test the strain limits of these sensors, several cycles were applied with different maximum strains, as depicted in Figure 10, using a constant strain speed. The signals obtained from the wavelength (Figure 10a) and the transmittance (Figure 10b) coordinates of the LSPR band minimum also offered a consistent optical response that seemed to saturate for a maximum strain of 13.4% (*ε* = 0.134). Nonetheless, the sensor seems to be recovering for all the used strains. Similarly to the tensile tests with speed variations in Figure 9, the transmittance coordinate of the LSPR band minimum has a superior signal-to-noise ratio than the wavelength coordinate signal. 

Furthermore, to compare the influence of the PDMS substrate and the TiO_2_ matrix deposited on the PDMS substrate, a maximum strain of 10.1% (*ε* = 0.101) and a strain speed of 70 µm/s was used to test them. Since these materials have no plasmonic extinction band, a transmittance near the LSPR band minimum (650 nm) was analyzed. The results can be observed in Figure 10b. The transmittance of the PDMS substrate (Figure 10b in blue) only varied a small fraction of the plasmonic sensor (less than 10%), most probably due to some scattering as a result of the elongation of the polymer along the tensile direction, and compression on the perpendicular direction. Regarding the TiO_2_ matrix deposited on PDMS, the transmittance variation (Figure 10b in purple) was considerably higher than for the PDMS substrate, but still lower than for the Au-TiO_2_ thin film, reaching about 60% of the change in transmittance of the plasmonic sensor.

To quantify and compare the response of the plasmonic sensor to different strains, similarly to the gauge factor that is used in piezoresistive sensors [73] and surface-enhanced Raman scattering (SERS) piezoplamonics [74], the transmittance gauge factor (*GF_T_*) was defined as follows:

(1) $$GF_{T}=\frac{\frac{T_{initial}-T_{strained}}{T_{strained}}}{\varepsilon },$$

where $T_{initial}$ and $T_{strained}$ are the transmittance coordinate at the LSPR band minimum before and after the application of a maximum strain ε.

Furthermore, the sensitivity of both the transmittance (*S_T_*) and the wavelength (*S_λ_*) signals of the LSPR band minimum were calculated using the following equations [43]:

(2) $$S_{T}=\frac{\Delta T}{\varepsilon },$$

(3) $$S_{\lambda }=\frac{\Delta \lambda }{\varepsilon },$$

where ΔT and Δλ are the variations of the transmittance and the wavelength coordinate of the LSPR band minimum, respectively, after an applied strain ε.

**Figure 11:**
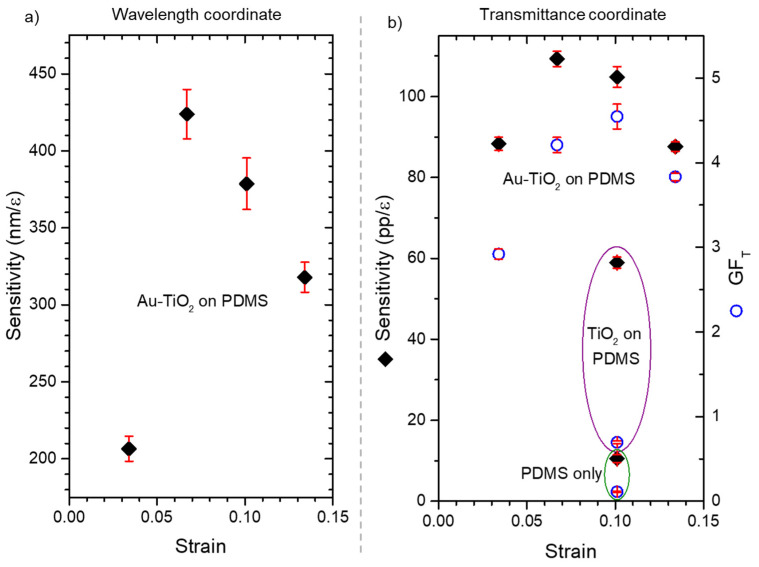
Sensitivity of (**a**) the wavelength and (**b**) the transmittance coordinates of the LSPR band minimum of the nanoplasmonic sensors, plotted against each maximum strain applied (3.4%, 6.7%, 10.1% and 13.4%). (**b**) Also shows the transmittance sensitivity (at 650 nm) of the PDMS substrate and a TiO_2_ thin film deposited on PMDS. Furthermore, the transmittance gauge factor (*GF_T_*) considering the transmittance coordinates of the nanoplasmonic sensor, the TiO_2_ on PDMS and the PDMS substrate are presented in (**b**).

The estimated sensitivities and gauge factors are represented in Figure 11, as a function of the maximum strain applied. It is obvious that, for both coordinates, the maximum sensitivity was reached for strains between of 6.7% (*ε* = 0.067) and 10.1% (*ε* = 0.101). At a strain of 6.7% the sensitivity for the wavelength coordinate reached 420 nm/*ε* (Figure 11a), while for the transmittance a sensitivity 110 pp/*ε* was achieved (Figure 11b). These results are close to those found in the literature for gold nanoparticles’ arrays [43], but, for this work, a change of the refractive index surrounding the nanoparticles seems to be the reversible event that tunes the LSPR band of the sensor for different applied strains.

When analyzing the transmittance gauge factor (Figure 11b) it is clear that the transmittance signal was enhanced in the plasmonic sensor, until a maximum strain of 10.1% (*ε* = 0.101) was attained, leading to a gauge factor of 4.5. The gauge factor of the PDMS substrate and the TiO_2_ thin film on PDMS, were considerably lower, 0.1 and 0.7 respectively. Therefore, when a strain of 10.1% was applied, the plasmonic thin film’s gauge factor was still 45 and 6.5 times higher than the gauge factor of PDMS and TiO_2_, respectively.

## 4. Conclusions

A novel flexible plasmonic strain sensor was prepared by functionalizing a PDMS substrate with a TiO_2_ thin film containing Au nanoparticles. Preliminary tensile tests revealed that the LSPR band of these sensors could be modified by applying different strains. After correlating these findings with the simulations results in different strain scenarios, it became plausible that, in these sensors, the LSPR band is adjusted mainly due to changes in the refractive index surrounding the Au nanoparticles, probably caused by some induced porosity. Several strain protocols were applied to the sensors with consistent repeatability, and the maximum sensitivity was achieved for strains between 6.7% and 10.1%.

## Data Availability

Raw data are available upon request to the corresponding author.

## Supplementary Materials

The following supporting information can be downloaded at: https://www.mdpi.com/article/10.3390/s22041375/s1, Figure S1: nanoDDSCAT+ software interface tools, Figure S2: Optical extinction efficiency coefficient estimation of several shapes with nanoDDSCAT+, Figure S3: Nanoparticles being compressed change their aspect ratios (AR = 1, AR = 1.4 and AR = 1.8) but keep the same effective volume., Figure S4: Array of 16 nm diameter nanoparticles being biaxially elongated, Figure S5: Array of 16 nm diameter nanoparticles being uniaxially elongated, Figure S6: Array of 16 nm diameter nanoparticles being uniaxially elongated with transverse compression.
