# Supplementary material for: Plasmonic Strain Sensors Based on Au-TiO2 Thin Films on Flexible Substrates
Source: Sensors (Basel). 2022 Feb 11;22(4):1375. doi: 10.3390/s22041375 (PMC8963073; doi:10.3390/s22041375)
Supplement: Supplementary file 1 [file sensors-22-01375-s001.zip › sensors-1572608-SI.pdf]

Plasmonic strain sensors based on Au-TiO_2 thin films on flexible substrates

Marco S Rodrigues*, Joel Borges, Filipe Vaz

CF-UM-UP, Centro de Física das Universidades do Minho e do Porto, Universidade do Minho, Portugal

* Correspondence: marcopsr@gmail.com , mprodrigues@fisica.uminho.pt

1. Optical simulations with gold nanoparticles using nanoDDSCAT+

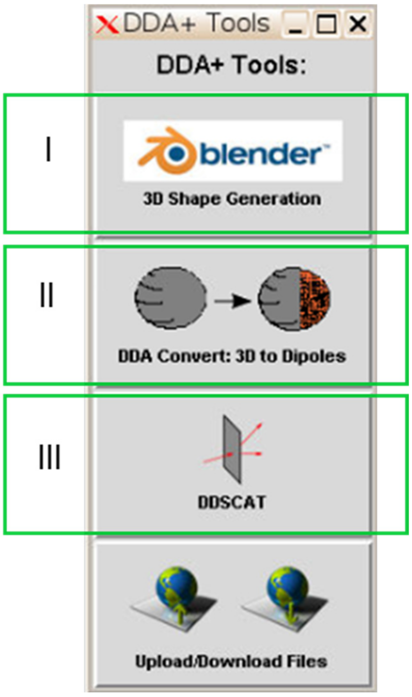

Figure S1. *nanoDDSCAT+ software interface tools, freely available at "nanohub.org". I - 3D shape generation (Blender tool); II - 3D shape to dipole conversion (DDA convert tool); III - Discrete dipole approximation code (DDSCAT tool).*

2. Individual nanoparticles

2.1. LSPR Extinction Band for Nanoparticles of Different Shapes

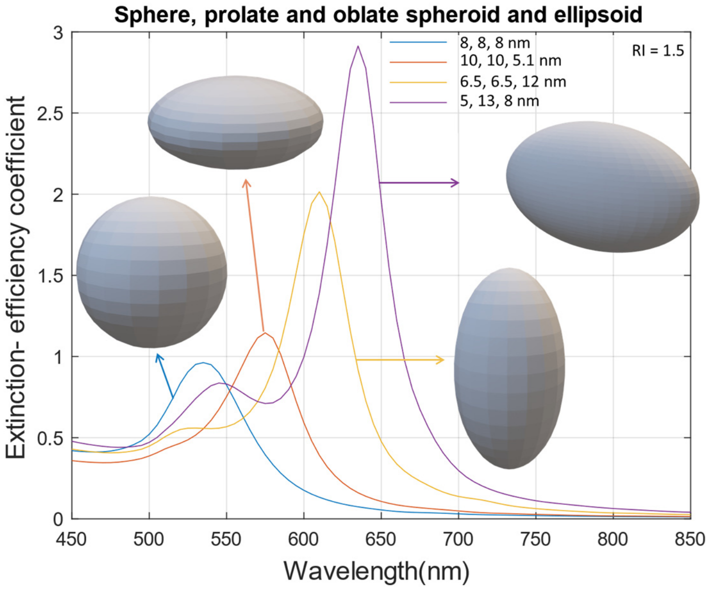

Figure S2. *Optical extinction efficiency coefficient estimation of several shapes with nanoDDSCAT+*

2.2. Compressing Single Nanoparticles

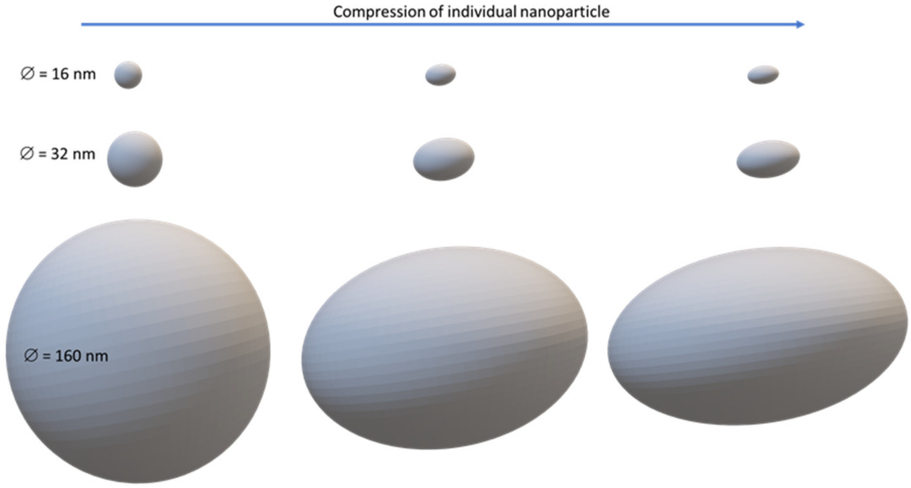

Figure S3. *Nanoparticles being compressed change their aspect ratios (AR = 1, AR = 1.4 and AR = 1.8) but keep the same effective volume.*

3. Elongation of a 2D nanoparticles network

3.1. Biaxial elongation of 16 nm gold nanoparticles network

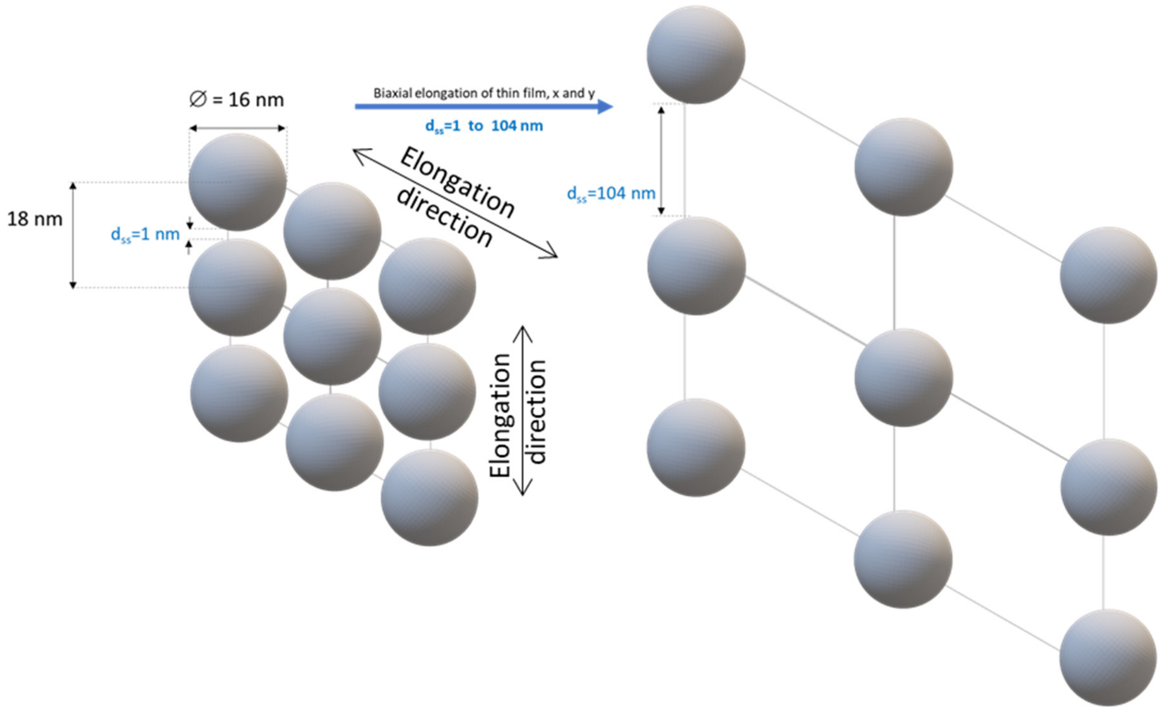

Figure S4. *Array of 16 nm diameter nanoparticles being biaxially elongated*

3.2. Uniaxial elongation of 16 nm gold nanoparticles network under different surrounding refractive indices.

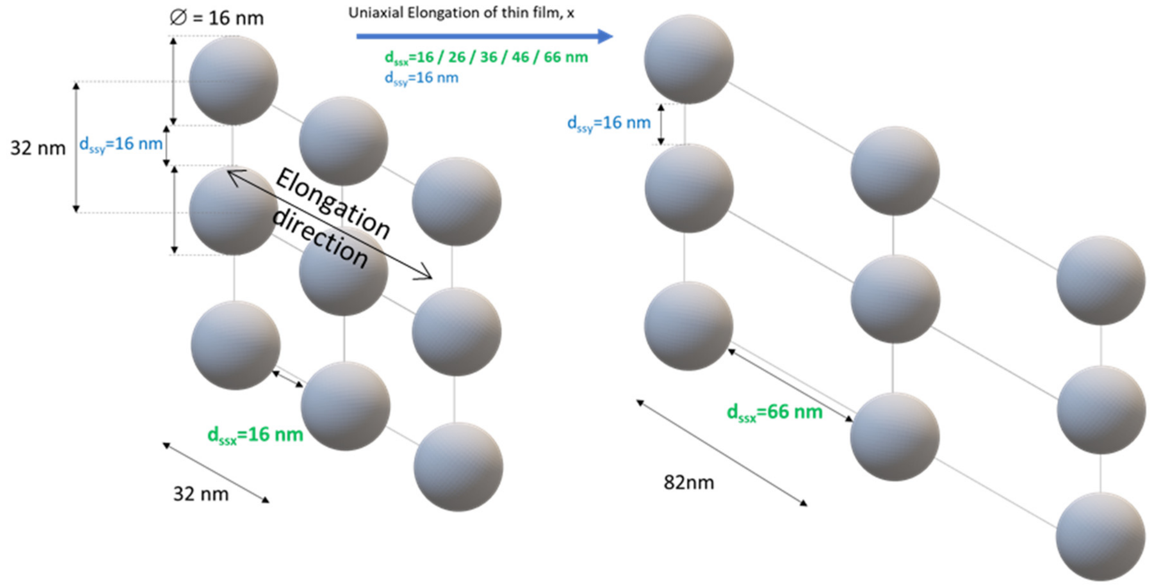

Figure S5. *Array of 16 nm diameter nanoparticles being uniaxially elongated*

3.3. Uniaxial elongation of 16 nm gold nanoparticles network with transverse compression.

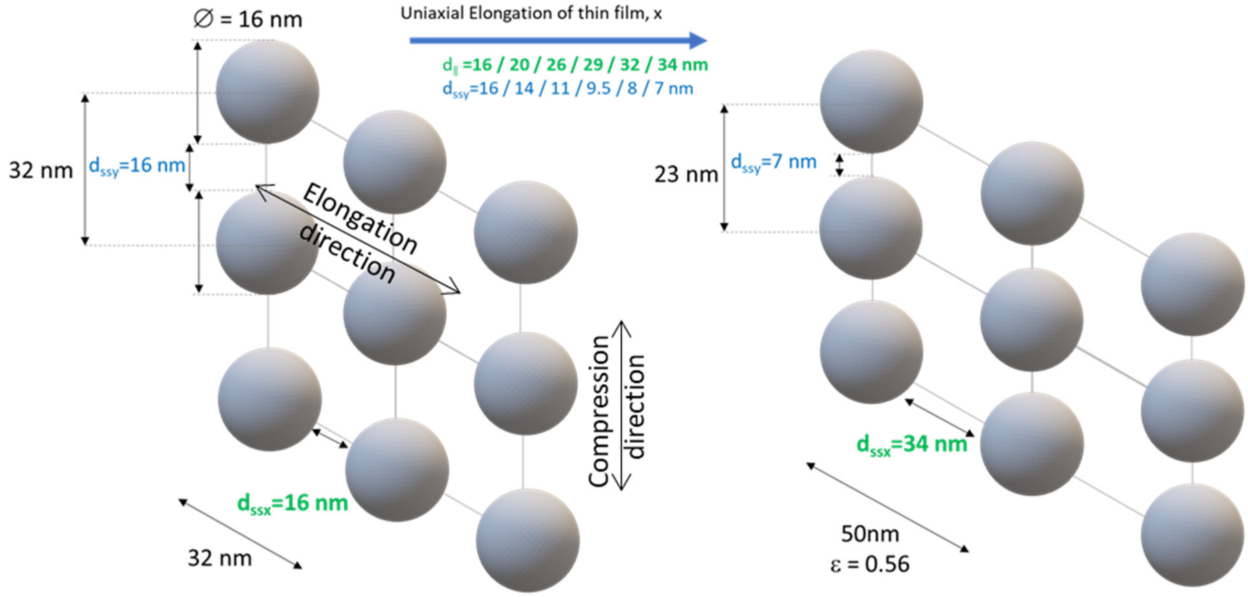

Figure S6. *Array of 16 nm diameter nanoparticles being uniaxially elongated with transverse compression*
